# Undifferentiated Pleomorphic Sarcoma and the Importance of Considering the Oncogenic and Immune-Suppressant Role of the Human T-Cell Lymphotropic Virus Type 1: A Case Report

**DOI:** 10.3389/fonc.2017.00091

**Published:** 2017-05-24

**Authors:** Sergio Lupo, Carolina Berini, Camila Cánepa, Eduardo Santini Araujo, Mirna Biglione

**Affiliations:** ^1^School of Medical Sciences (UNR) and UAI Rosario, Centralized Institute of Integral Clinical Research (CAICI), Rosario, Argentina; ^2^CONICET-Universidad de Buenos Aires, Instituto de Investigaciones Biomédicas en Retrovirus y SIDA (INBIRS), Buenos Aires, Argentina; ^3^Laboratory of Orthopaedic Pathology, Buenos Aires, Argentina

**Keywords:** sarcoma, HTLV-1, mutation, myelolpathy, immunodeficiency

## Abstract

**Introduction:**

Soft-tissue sarcomas account for 0.7% of all malignant tumors, with an incidence rate of 3 per 100,000 persons/year. The undifferentiated pleomorphic sarcoma (UPS) with giant cells, a high grade tumor of soft tissue, is very unusual, especially in young adults before the age of 40. Human T-cell lymphotropic virus type 1 (HTLV-1) is a human retrovirus, classified as group 1 human carcinogens by The International Agency for Research on Cancer, that causes an aggressive malignancy known as adult T-cell lymphoma/leukemia and a progressive chronic inflammatory neurological disease named HTLV-1-associated myelopathy/tropical spastic paraparesis (HAM/TSP). HTLV-1 causes accumulation of genetic mutations in the host genome that could contribute to cellular transformation, one of the oncogenic features of HTLV-1.

**Case report:**

We describe a case of a young woman with UPS who suffered from HAM/TSP with 3 years of evolution. In 2013, the patient started with neurological symptoms: weakness in the legs and bladder dysfunction. One year later, the patient developed a mild paraparesis in both extremities, anti-HTLV-1 antibodies were detected in plasma and in cerebrospinal fluid, and HAM/TSP was confirmed. In November 2015, a benign ganglion cyst was first suspected without intervention and by March 2016 a sarcoma was diagnosed. Three weeks after surgical resection, the tumor aroused in deep tissue and behaved aggressively, implicating a curative wide resection of the fibula, joint reconstruction, and soft-tissue graft. Histopathological examination confirmed UPS with giant cells.

**Concluding remarks:**

The unapparent subclinical immunodeficiency state due to HTLV-1 infection deserves to be considered in order to carefully monitor the possibility of developing any type of cancer. Besides, reaching an accurate and timely diagnosis of UPS can be challenging due to the difficulty in diagnosis/classification and delayed consultation. In this particular case, considering the high grade of UPS and the progressive invalidating myelopathy caused by HTLV-1, treatment should be carefully evaluated to positively impact on the patient’s life expectancy.

## Introduction

Human T-cell lymphotropic virus type 1 (HTLV-1) is a retrovirus that causes an aggressive malignancy known as adult t-cell lymphoma/leukemia (ATLL) and a progressive chronic inflammatory neurological disease named HTLV-1-associated myelopathy/tropical spastic paraparesis (HAM/TSP). In 2014, HTLV-1 was classified as group 1 human carcinogens by The International Agency for Research on Cancer (IARC) (1). It was demonstrated that the oncoprotein HTLV-1 Tax carries out essential functions in order to deregulate cellular division. Tax can inhibit multiple DNA repair pathways and stimulate incorrect DNA repair allowing accumulation of genetic mutations in the host genome and contributing to cellular transformation. This mechanism has recently been named by Nicot et al. as “Random Mutagenesis Model” to characterize one of the oncogenic features of HTLV-1 (2).

Considering undifferentiated pleomorphic sarcoma (UPS), two missense mutations in the *KRAS* proto-oncogene and in *PIK3CA* have been recently identified by gene mutation screening (3). *KRAS* (located at 12p12.1) is frequently altered with mutations occurring in 17–25% of all cancers, while mutations in *PIK3CA* have been identified in several human solid tumors including breast, colon, ovarian, liver, and lung cancers (4, 5).

## Case Report

We present a case of UPS with giant cells in a 46-year-old woman with HAM/TSP from a non-endemic HTLV-1 area of Argentina. In November 2013, the patient started with weakness in the legs and bladder dysfunction. One year later, she presented mild paraparesis in both extremities and hyperreflexia. By February 2015, detection of anti-HTLV-1 antibodies in plasma and cerebrospinal fluid (CSF) by ELISA (HTLV I&II Ab, ULTRA version, Dia.Pro) and western blot (HTLV Blot 2.4, MP Diagnostics) confirmed HTLV-1 infection. For HTLV-1 molecular confirmation and proviral load (PVL), DNA was extracted from peripheral blood mononuclear cells (PBMCs) by column extraction (ADN PuriPrep-S kit, Highway^®^, Inbio, Tandil, Argentina) and analyzed with “in-house” n-PCR and by real-time SYBR Green PCR (qPCR) as previously described, respectively (6, 7). HTLV-1 *pol* and *tax* regions were detected and PVL determined a total of 7.9 copies of HTLV-1/100 PBMC. No other pathologies have been found through imaging, blood and CSF examination and HAM/TSP diagnosis was confirmed. By June 2015, difficulty in walking due to spasticity progressed and PLV value was 17.3 copies of HTLV-1/100 PBMC; an antiretroviral treatment (tenofovir and emtricitabine) was indicated with a decreased in PVL in the next months and improvement of neurological symptoms and signs.

**Figure 1:**
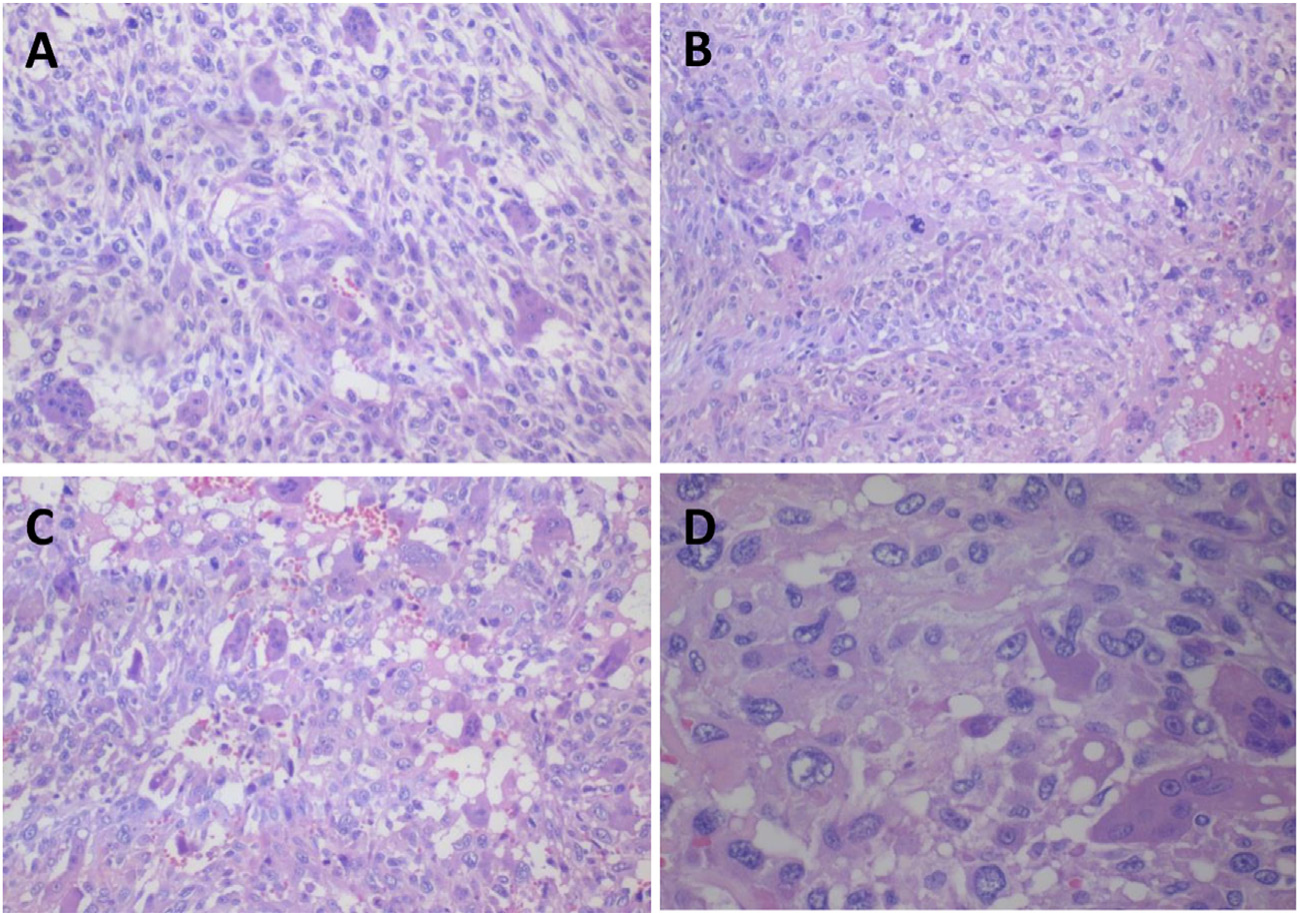
**Representative histologic section of undifferentiated pleomorphic sarcoma with giant cells obtained from the anterior right ankle mass**. **(A)** Osteoclastic-like multinucleated cells mixed with pleomorphic and predominantly atypical round cell population (H–E, ×100), **(B)** Multinucleated giant cells admixed with tumor pleomorphic cells with atypical mitosis (H–E, ×100), **(C)** Hemorrhagic areas (H–E, ×100), and **(D)** Osteoclastic giant cells with irregular nuclei and prominent nucleoli. The pleomorphic cells have much longer nuclei (H–E, ×200).

In November 2015, the patient attended for the first time a dermatology service due to a cutaneous painless mass, soft in consistency (diameter <5 cm) that had arisen in the anterior right ankle. A benign ganglion cyst was first suspected without the need of an immediate intervention. However, a consistent mass grew quickly. A computed tomography revealed a mass of indefinite borders, and consequently, a surgery to remove the tumor was performed in March 2016. Based on a biopsy, a sarcoma was diagnosed; metastasis was discarded by positron emission tomography (PET) scans. Three weeks after surgical resection of the primary tumor, a new mass was detected near the primary one and the patient attended a private hospital, where a local recurrence was confirmed. Metastasis was again discarded by PET scans. She had no pain, no symptoms (weight loss and/or fatigue), and the laboratory analysis was normal. In June 2016, after a new laboratory exam and MRI, two possible surgical interventions were discussed in an athenaeum with different specialists: leg amputation considering the rapid progressive spasticity due to HAM/TSP or an excisional biopsy. The latter was indicated considering also the patient’s decision. This time the tumor aroused in deep tissue and behaved aggressively, implicating a complete and extended surgical resection to the fibula, joint reconstruction, and soft-tissue graft. Macroscopic examination showed a tumor with a solid grayish cut surface and no clear envelope. The histopathology exam showed fascicles and sheets of spindle cells in a loose/storiform pattern. Numerous markedly atypical cells and some multinucleated giant cells admixed with tumor pleomorphic cells were observed. Chronic inflammation and abundant atypical mitosis, and necrosis, and hemorrhages areas were also present. This histopathologic pattern defines the lesion as UPS with giant cells (Figure 1). A panel of immunohistochemistry was performed expressing Vimentin, focal smooth muscle actin, and CD34 being negative for Desmin, S100, CK AE1, AE3, and CK18. Complete resection with negative margins was also observed and radiotherapy was indicated. Later, HTLV-1 integration was determined in biopsy cells by nested-PCR and qPCR (PVL was 0.74 copies/PBMC).

In September 2016, the patient had to begin chemotherapy due to bilateral pulmonary metastasis [doxorubicin, ifosfamide, and mesna repeated every 21 days as recommended by the European Organization and Treatment of Cancer Soft Tissue and Bone Sarcoma Group (8)]. By February 2017, the patient had already finished the treatment and showed a favorable response. In April, two local recurrences were detected and amputation of the leg was indicated.

## Background and Discussion

Soft-tissue sarcomas account for 0.7% of all malignant tumors, with an incidence rate of 3 per 100,000 persons/year. UPS, formerly called “malignant fibrous histiocytoma,” is an entirely new category of tumors, introduced for the first time in the 2013 classification, which are diagnosed in just a few thousand cases each year (9, 10). UPS comprises a series of high-grade soft-tissue sarcomas often associated with poor prognosis. This new category comprises up to 20% of all pleomorphic soft-tissue sarcomas including radiation-associated sarcomas and tumors in which all recognizable lines of differentiation have been excluded (10, 11). UPS appears typically in patients that are approximately 50–70 years of age, with a slight male predominance and can arise in any part of the body, but most commonly in the extremities. Local recurrence of the tumor in the same location occurs in 25% of all the patients with soft-tissue sarcomas but it is lower in soft-tissue extremity sarcomas. Most recurrences develop in the first 2 years after treatment but can occur at any time during the life of the patient. Superficial tumors have better prognosis and two-thirds recur. Nonetheless, metastases are infrequent. Deep tumors recur in about 40%, and 50% of them metastasize. The most frequent metastatic presentation is pulmonary disease (90%) being extra-pulmonary sites uncommonly involved lymph nodes (10%), bone (8%), and liver (1%) (12).

Human T-cell lymphotropic virus type 1 is a human retrovirus that was isolated for the first time from lymphocytes of a patient with cutaneous T-cell lymphoma in 1980 (13). HTLV-1 is distributed worldwide, with areas of high endemicity close to others without the presence of infection. The highest HTLV-1 prevalence is found in Southwestern Japan, sub-Saharan Africa and South America, the Caribbean area, and some regions in Middle East and Austral-Melanesia. In non-endemic areas, the virus is circulating with higher prevalence among vulnerable populations or in reference to immigrants or ancestors from endemic areas. Similar to HIV, this retrovirus is transmitted from mother to child and by sexual and parenteral route. HTLV-1 prevalence increases gradually with age, especially among women in endemic areas. Approximately 10 million individuals are infected with HTLV-1 worldwide, and of them, 1–5% will develop an associated pathology (14). HTLV-1 is a human retrovirus capable of causing cancer, and it has been recently classified as group 1 human carcinogens by The IARC (1). It causes an aggressive malignancy known as ATLL and a progressive chronic inflammatory neurological disease named HTLV-1-associated myelopathy/tropical spastic paraparesis (HAM/TSP) that is progressively invalidating (14). In this case, sexual transmission from her husband was confirmed, who got infected from her mother who died of ATLL years before in a non-endemic area of Argentina (15). So, physicians should bear HTLV-1 infection in mind and suspect it regardless of the patient’s residence.

HAM/TSP is characterized for producing a chronic inflammation in the central nervous system with an insidious onset and slow progressive evolution. Both serum and CSF present elevated levels of several proinflammatory cytokines (16). Also, perivascular inflammatory cell infiltration with demyelination focused on the thoracic spinal cord is observed. Neurological manifestations include spastic paraparesis, sphincter dysfunction, and sensory impairment. According to Osame criteria (17), HAM/TSP diagnosis is based on clinical findings and demonstration of anti-HTLV-1 antibodies in serum and CSF (17).

Human T-cell lymphotropic virus type 1 presents tropism for CD4-positive lymphocytes from PBMC. It has been described that the number of CD4-infected cells could increase just after infection and then decreases in a year to keep them constant for more than 10 years. In this context, clonal expansion of infected cells would be responsible for the maintenance of HTLV-1 infection. The expression of certain clones of Tax could develop and differentiate in a phenotype able to avoid immune surveillance. If some of these clones accumulate genomic abnormalities, they can develop a pre-leukemic state in some individuals (18). On the other hand, the increasing numbers in certain T-cells due to HTLV-1 infection may also cause imbalance of the immune system, resulting in immune dysfunction or inflammatory diseases such as myelopathy and uveitis (19).

There are no cases reported in the literature of UPS linked to HTLV-1 infection. However, there have been several reports on HTLV-1 infection in mice in which clonal proliferation of HTLV-1 has been associated with spontaneous malignant tumor formation (20). Regarding these data, histiocytic sarcoma invasion into the lumbar spinal cord has been described in transgenic mice that developed HAM/TSP-like disease supporting the oncogenic feature of HTLV-1 (1, 21). In this case, the patient had no laboratory results or symptoms related to an immunocompromised status even though it should not be discarded due to HTLV-1 infection which could contribute to UPS oncogenesis by both, direct and indirect mechanisms.

It has been reported that some sarcomas usually show a complex genomic profile, some of which present a frequent loss of RB1 and alterations of p53 (22). In this context, Johnson et al. reported that Tax affects cell-cycle regulatory molecules, stabilizes, and inactivates the tumor suppressor p53 in HTLV-1 transformed cell lines by inducing hyperphosphorylation of p53 (19). Considering the “Random Mutagenesis transformation model” proposed to characterize the oncogenic activities of HTLV-1 (2), we could hypothesize that mutations linked to the UPS could have likely been produced in this case. Further research on gene–gene and gene–environment interactions of this oncogenic virus is need.

## Concluding Remarks

This case describes a rare outcome of an undifferentiated sarcoma with giant cells in a HAM/TSP Caucasian patient in which HTLV-1 integration was detected in biopsy cells. Even though the role of HTLV-1 in this type of cancer is uncertain, its oncogenic and immune suppressor role should be particularly considered. Consequently, the possibility for patients to develop any type of cancer must be bearded in mind to reach an accurate and timely diagnosis of tumors. Given the high grade of UPS and the progressive invalidating myelopathy caused by HTLV-1, the treatment should be carefully evaluated to positively impact on the patient’s life expectancy. On the other hand, training on HTLV-1 pathologies should be implemented among physicians to avoid time and anguish in the patient to obtain a correct diagnosis particularly in non-endemic areas.

## Ethics Statement

The patient agreed and provided written informed consent for publication of this case report and any accompanying imaged. Due to the observational nature of this case report, no formal ethics approval was required.

## Author Contributions

Study concept design: MB, SL, and ESA. Formal analysis and interpretation of data; drafting of the manuscript: SL, CB, CC, and MB. Resources: SL and ESA. Critical revision of the manuscript for important intellectual content: MB, SL, CB, and ESA. All the authors agreed to be accountable for all aspects of the work in ensuring that questions related to the accuracy or integrity of any part of the work are appropriately investigated and resolved.

## Conflict of Interest Statement

The manuscript is not under active consideration for publication, has not been accepted for publication, nor has it been published, in full or in part (except in abstract form). All co-authors had access to all the study data and were responsible for the accuracy of the analysis. They also had authority over manuscript preparation and the decision to submit the manuscript for publication. The authors declare that the research was conducted in the absence of any commercial or financial relationships that could be construed as a potential conflict of interest.
